# Modeling of Millimeter-Wave Modulation Characteristics of Semiconductor Lasers under Strong Optical Feedback

**DOI:** 10.1155/2014/728458

**Published:** 2014-10-14

**Authors:** Ahmed Bakry

**Affiliations:** Department of Physics, Faculty of Science, King Abdulaziz University, Jeddah 21589, Saudi Arabia

## Abstract

This paper presents modeling and simulation on the characteristics of semiconductor laser modulated within a strong optical feedback (OFB-)induced photon-photon resonance over a passband of millimeter (mm) frequencies. Continuous wave (CW) operation of the laser under strong OFB is required to achieve the photon-photon resonance in the mm-wave band. The simulated time-domain characteristics of modulation include the waveforms of the intensity and frequency chirp as well as the associated distortions of the modulated mm-wave signal. The frequency domain characteristics include the intensity modulation (IM) and frequency modulation (FM) responses in addition to the associated relative intensity noise (RIN). The signal characteristics under modulations with both single and two mm-frequencies are considered. The harmonic distortion and the third order intermodulation distortion (IMD3) are examined and the spurious free dynamic range (SFDR) is calculated.

## 1. Introduction

The radio over fiber (RoF) technology has recently attracted considerable attention as an integration of wireless and optical systems and consequently as a solution to enhance the communication bandwidth to support integrated services [1]. This technology inherently combines the advantage of enormous bandwidth of optical fiber and the flexibility of wireless access technologies to deliver wireless RF signals directly from the central station to simplified base stations. The mm-wave bands are utilized to meet the demand for higher signal bandwidth and to overcome the frequency jamming in the RoF-based wireless networks [2]. However, enhancing the bandwidth of RoF links with directly modulated semiconductor lasers to the mm-wave band is limited by the available modulation bandwidth of semiconductor lasers. Increasing the differential gain is an efficient technique to increase the modulation bandwidth [3]. Modulation up to a frequency of 25 GHz was achieved [4]; however a record of 40 GHz response is challenging. In addition, at frequencies near the relaxation oscillation frequency, the laser noise and distortion increase [5]; therefore the usable bandwidth for directly modulated analog links is less than the possible 3 dB bandwidth.

The technique of injection locking has been reported by several groups to enhance the modulation bandwidth of laser diodes [6–8]; high-frequency modulation beyond 40 GHz was demonstrated [9, 10]. External OFB has been shown to be an alternative and cost-effective technique to increase the modulation bandwidth of semiconductor lasers, depending on appropriate choices of the system parameters [11–16]. Narrow-band high-frequency modulation over 40 GHz has been achieved in quantum well lasers under OFB [17]. Semiconductor lasers with OFB display rich chaotic dynamic behaviors, including period-1 oscillations, period doubling, quasiperiod, and routes to chaos due to variations in the phase of the reinjected field into the laser cavity [15, 18]. Under strong OFB, the frequency of the induced oscillations could be comparable to a resonance frequency of the external cavity [19]. Most recently the author's group [20] has newly reported using strong OFB to boost the modulation frequencies over an ultrahigh frequency passband over 55 GHz and has shown improvement of the gain of a corresponding RoF link by about 20 dB. Such enhancement in the IM response over an ultrahigh frequency passband was attributed as a type of photon-photon resonance due to coupling of oscillating modes in the coupled cavity [13, 21, 22]. It is obtained when the nonmodulated laser keeps operation in CW under strong OFB. The authors pointed out also that the noise factor of such mm-wave RoF links improves nearly by 20 dB in the regime of small-signal modulation and 10 dB under large-signal modulation [23].

In this paper, we introduce comprehensive investigation on the mm-wave modulation characteristics and noise of semiconductor lasers with a short-external cavity and strong OFB. We newly discuss the FM response and the frequency chirp associated with this ultrahigh speed IM. Because the signal distortion and the dynamic range of the laser are critical issues in the modulation of semiconductor lasers and the optical analog links [24, 25], we also examine the harmonic distortions in the mm-wave modulated laser signal. We consider modulation of the laser with single mm-frequency and study the associated second order harmonic distortion (2HD). We study also the modulation performance of the laser modulation using two adjacent mm-frequencies and measure the associated IMD3 and the corresponding SFDR. The present study is based on applying a strong OFB rate equation model, in which OFB is treated as time delay of OFB with roundtrips (multiple reflections) in an external cavity [4]. The noise content of the mm-wave modulated signal is characterized by the frequency spectrum of RIN. The SFDR, which is defined as the dynamic range at the modulation power when the system noise floor is equal to the distortion noise, is measured by the power of the fundamental frequency component, noise floor, and the power in the IMD3 component [26]. We compare the obtained findings with those of a solitary laser when modulated at the carrier-photon resonance (relaxation) frequency, which is the most practical frequency regime of the solitary laser at which the IM is most enhanced. We apply the model to a high-speed DFB laser with a modulation bandwidth of about 25 GHz [4]. We show that, compared with the solitary laser modulated at the relaxation frequency, the present modulated signal was shown to have 5 dB lower distortions, 10 dB/Hz lower RIN, and 2 dB/Hz^3/2^ higher SFDR.

In the next section, we introduce the time-delay rate equation model of analyzing OFB with sinusoidal modulation and the associated noise. The numerical procedures of the present model are given in Section 3. In Section 4, we present the simulated IM and FM responses and noise characteristics under single- and two-tone modulations. Finally, the present work is concluded in Section 5.

## 2. Theoretical Model

The dynamics and noise of semiconductor under both IM and external OFB are described by the following time-delay rate equations of the carrier number *N*(*t*), photon number *S*(*t*), and optical phase *θ*(*t*) [23]:

(1) $$\begin{array}{c} \frac{dN}{dt}=\frac{1}{e}I\left(t\right)-\frac{N}{\tau_{s}}-\frac{av_{g}}{V}\frac{N-N_{g}}{1+\varepsilon S}S+F_{N}\left(t\right), \end{array}$$

(2) $$\begin{array}{c} \frac{dS}{dt}=\left[\Gamma \frac{av_{g}}{V}\frac{N-N_{g}}{1+\varepsilon S}-G_{\text{th}}\right]S+C\frac{N}{\tau_{s}}+F_{S}\left(t\right), \end{array}$$

(3) $$\begin{array}{c} \frac{d\theta }{dt}=2\pi \Delta \nu \left(t\right)=\frac{1}{2}\left(\alpha \Gamma \frac{av_{g}}{V}\left(N-N_{\text{th}}\right)-\frac{v_{g}}{L_{D}}\varphi \right)+F_{\theta }\left(t\right), \end{array}$$

where Δ*n*(*t*) is the frequency chirp induced by the instantaneous variation of the optical phase due to variation in *S*(*t*) and *N*(*t*). In (2), *G*_th_ is the threshold gain under OFB and is determined by the photon lifetime *τ*_*p*_ in the laser cavity of length *L*_*D*_ and refractive index *n*_*D*_,

(4) $$\begin{array}{c} G_{\text{th}}=\frac{1}{\tau_{p}}-\frac{v_{g}}{L_{D}}\ln\left|U\left(t-\tau \right)\right|. \end{array}$$

In the above equations, *a* is the differential gain coefficient, *v*_*g*_ is the group velocity in the active layer of length *L*_*D*_, Γ is the confinement factor, *α* is the linewidth enhancement factor, *τ*_*s*_ is the spontaneous emission lifetime, *ε* is coefficient of gain suppression, *N*_*g*_ is the electron number at transparency, and *N*_th_ is the electron number at threshold. In (4), *U*(*t* − *τ*) is an OFB function that describes the time delay of laser radiation due to roundtrips (i.e., multiple reflections) in the external cavity (of length *L*_ex_ and refractive index *n*_ex_) formed between the laser front facet (of reflectivity *R*_*f*_) and the external mirror (*R*_ex_) [27, 28],

(5) U(t−τ)=|U(t−τ)|e−jφ =1−∑p=1(Kex)p(Rf1−Rf)p−1e−jωτS(t−pτ)S(t)ejθ(t−pτ)ejθ(t),

(6) φ=−tan−1Im⁡{U(t−τ)}Re{U(t−τ)}+nπ n:integer

with *ω* being the angular frequency of the laser emission and *τ* = 2*n*_ex_ *L*_ex_/*c* as the roundtrip time. The strength of OFB is measured by the coupling coefficient *K*_ex_, which is determined by the ratio between *R*_ex_ and *R*_*f*_ [27, 28]:

(7) $$\begin{array}{c} K_{\text{ex}}=\left(1-R_{f}\right)\sqrt{\eta \frac{R_{\text{ex}}}{R_{f}}}, \end{array}$$

where *η* is the external coupling efficiency of the injected light into the laser cavity. In (6) *n* is an integer and is chosen to vary continuously for time evolution, because the solution of arc tangent is limited in the range of −*π*/2 to *π*/2 in the computer work. At a given time *t*, the phase difference between the time-delayed (externally injected) field and the field inside the laser cavity is given by *θ*(*t* − *mτ*) − *θ*(*t*), which is equal to zero or *π* in the cases of in-phase and out-of-phase conditions.

The injection current *I*(*t*) is composed of a bias component *I*_*b*_ and a sinusoidal component of amplitude *I*_*m*_ and frequency *f*_*m*_:

(8) $$\begin{array}{c} I\left(t\right)=I_{b}+I_{m}\sin\left(2\pi f_{m}t\right). \end{array}$$

The modulation depth is given as *m* = *I*_*m*_/*I*_*b*_. The last terms *F*_*N*_(*t*), *F*_*S*_(*t*), and *F*_*θ*_(*t*) in rate equations (1)–(3) are Langevin noise sources with zero mean values and are added to the equations to account for intrinsic fluctuations of the laser [28]. These noise sources are assumed to have Gaussian probability distributions and to be *δ*-correlated processes [28]. The frequency content of intensity fluctuations is measured in terms of RIN, which is calculated from the fluctuations $\delta S\left(t\right)=S\left(t\right)-\bar{S}$ in *S*(*t*), where $\bar{S}$ is the time-average value of *S*(*t*). Over a finite time *T*, RIN is given as [29]

(9) $$\begin{array}{c} \text{RIN}=\frac{1}{{\bar{S}}^{2}}\left\{\frac{1}{T}{\left|\overset{T}{\underset{0}{\int }}\delta S\left(t\right)e^{-j2\pi f\tau }d\tau \right|}^{2}\right\}, \end{array}$$

where *f* is the Fourier noise frequency.

## 3. Numerical Calculations

Rate equations (1)–(3) are solved numerically by the 4th order Runge-Kutta method using a time integration step as short as 0.2 ps to allow simulation of the very high speed modulated signal. Five roundtrips, *p* = 1 → 5, are counted in the calculations. At each integration instant, the noise sources *F*_*N*_(*t*), *F*_*S*_(*t*), and *F*_*θ*_(*t*) are generated following the technique developed in [30] using a set of three uniformly distributed random numbers generated by the computer. In the simulations, we use the numerical values listed in Table 1, which correspond to single-mode quantum-well DFB laser [27]. This laser has a threshold current of *I*_th_ = 10 mA. The laser is assumed to be biased above threshold, *I*_*b*_ = 5*I*_th_. We adjust the length of the external cavity to be *n*_ex_ *L*_ex_ = 0.25 cm, which corresponds to an external-cavity resonance frequency spacing ~60 GHz. The fast Fourier transform (FFT) is used to simulate the frequency content of the modulated laser signal. The IM response and the associated FM response are calculated numerically, respectively, as

(10) $$\begin{array}{c} \text{IM}-\text{repsonse}=\frac{a_{1}\left(f_{m}\right)}{a_{1}\left(f_{m}⟶0\right)}, \end{array}$$

(11) $$\begin{array}{c} \text{FM}-\text{response}=\frac{b_{1}\left(f_{m}\right)}{I_{m}}, \end{array}$$

where *a*_1_(*f*_*m*_) and *b*_1_(*f*_*m*_) are the fundamental harmonics of the FFT spectra of the laser intensity and frequency at the modulation frequency *f*_*m*_.

## 4. Results and Discussion

### 4.1. Modulation Response of the Solitary Laser

**Figure 1:**
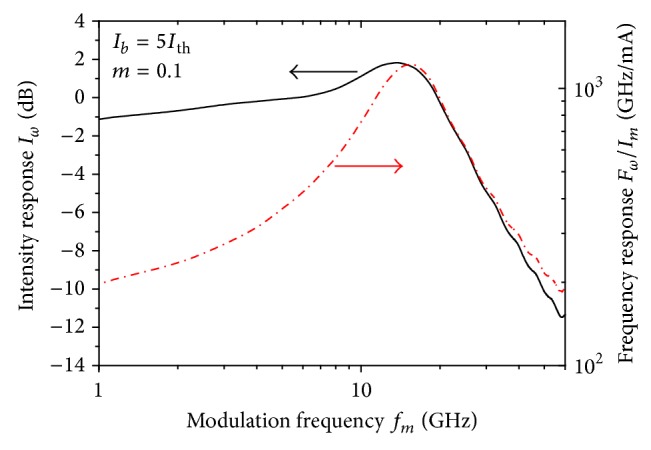
The intensity and frequency modulation responses of the solitary laser when *I*_*b*_ = 5*I*_th_ and *m* = 0.1.

Analog modulation of the injection current leads to variation in the injected carrier density into the active region, which results in a variation of the laser frequency.  Figure 1 plots both the simulated IM and FM responses of the solitary laser for when the modulation is as weak as the modulation depth which is *m* = 0.1. The FM response has a maximum at the relaxation frequency *f*_*r*_ ~ 15 GHz, which is slightly above the IM response at *f*_*p*_ = 14 GHz [31]. The IM response has a 3 dB-modulation bandwidth of *f*_3 dB_ = 25 GHz. The modulation response spectrum can be understood as follows [32]. When *f*_*m*_ is much lower than *f*_*r*_, the injected carriers follow the change in the injection current resulting in a flat response. Around the resonance frequency, the charge carriers interact with the photons with phase synchronization, which results in the laser resonance and the peaked response. The declining part of the modulation response is because the phase of the photon field lags behind that of the injection current. When *f*_*m*_ increases beyond *f*_*r*_, the electron and photon fields tend to become more and more out of phase, resulting in damping of the relaxation oscillations and reduction in the IM response.

### 4.2. Modulation Response under OFB

**Figure 2:**
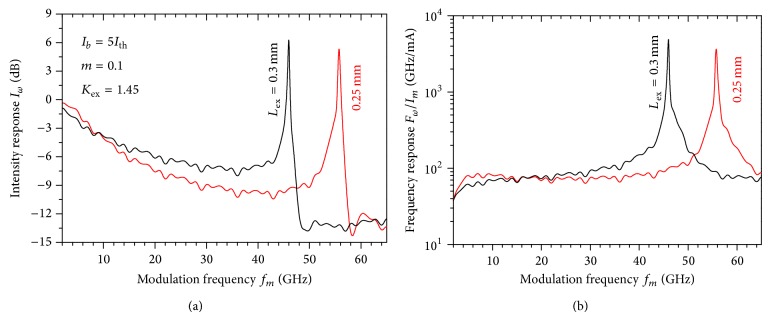
The intensity modulation responses of the laser under OFB with *K*_ex_ = 1.45 when *L*_ex_ = 0.25 and 0.30 mm with *m* = 0.1.

The present case of a semiconductor laser with a short cavity is characterized by a frequency ratio *f*_ex_/*f*_*r*_ > 1, which corresponds to a period-doubling route-to-chaos [18, 33, 34]. In the regime of strong OFB, the frequency of the possible oscillations may reach the external-cavity resonance frequency *f*_ex_ = *n*_ex_ *L*_ex_/*c*. In Figure 2(a), we plot examples of the numerical IM responses of the laser under strong OFB that are characterized by resonance enhancement over a mm-waveband. In Figure 2(b), we plot the corresponding FM responses. The shown IM responses are simulated for two short-external cavities with lengths of *L*_ex_ = 0.25 and 0.30 mm, which correspond to external-cavity resonance frequencies of 60 and 30 GHz, respectively. In this case the modulation depth is *m* = 0.1 and the OFB is as strong as *K*_ex_ = 1.45. Our simulation showed that the nonmodulated laser diode operates in CW in this level of OFB, where the injected delay light is nearly in phase with the optical field in the laser cavity. The figure shows that the IM response drops under the –3 dB level at the frequencies of *f*_*m*_ = 8 and 6 GHz when *L*_ex_ = 0.25 and 0.30 mm, respectively, which are much lower than *f*_3 dB_  of the solitary laser. In the high-frequency regime the IM response is enhanced over the mm-wave bassbands of (54.4 and 56.5 GHz) and (45 and 46.6 GHz) centered at the frequencies *f*_*m*_ = 55.8 and 46 GHz when *L*_ex_ = 0.25 and 0.30 mm, respectively. These frequency bands are much higher than *f*_3 dB_ of the solitary laser. The IM enhancement over the IM response of the solitary laser is as large as 5.4 dB and 6.5 dB, respectively, which may be due to higher degree of phase-matching between the coupling OFB and the optical field in the laser cavity. Similar behavior of the narrow-band enhancement of the IM was reported by Troppenz et al. [16] around 40 GHz. Figure 2(b) shows that, contrary to the case of the solitary laser, the peaks of the FM responses occur at the same peak frequencies of the IM responses. The FM responses are lower than that of the solitary laser at frequencies lower than the mm-frequency passbands but are enhanced within these frequency bands. The amplitudes of these FM responses are ~3.2 and 5.0 times larger than that of the solitary laser when *L*_ex_ = 0.25 and 0.30 mm, respectively.

This mm-narrow band enhancement of the modulation response can be attributed to coupling between the resonance modes of the external cavities because of the carrier pulsation in the laser cavity at the beating frequency *f*_ex_. This carrier pulsation is induced by the modulating current signal *I*(*t*) as indicated by (8) and the rate (1) of the injected carrier number *N*(*t*). This resonance is induced by optical modes and is different from the conventional carrier-photon resonance, which occurs around the relaxation frequency of the laser. Therefore, this resonance is referred to as “photon-photon resonance” [13]. Similar effect is observed in vertical-cavity surface-emitting lasers (VCSELs) coupled to a transverse cavity [22], in which the photon-photon resonance is induced by transverse oscillating modes. Because the shown IM responses have an unused frequency between the frequency *f*_3 dB_ and the enhanced mm-narrow bands, this modulation enhancement is not favored for applications in telecommunications. It is interesting for applications such as the mm-wave RoF networks that require only a narrow bandwidth centered at a millimeterwave.* To recover the modulation response over this unused gap and achieve flat IM responses, dispersive techniques may be needed at the end mirrors to obtain and allocate the photon-photon resonance at mm-frequency and achieve a wide carrier-photon resonance [21].*

### 4.3. Modulation Performance in the mm-Frequency Band

As shown above, when the laser is subjected to strong OFB, the modulation frequency is enhanced over a narrow mm-frequency band that can be very close to the external cavity resonance frequency *f*_ex_. In this section, we characterize the laser modulation at the peak-frequencies of *f*_*m*_ = 55.8 and 46 GHz, which correspond to the external-cavity lengths of 0.25 and 0.30 mm, respectively, and compare the results with the modulation characteristics of the solitary laser when modulated at the carrier-photon resonance frequency *f*_*r*_. These characteristics include the waveforms of the signal power *P*(*t*) and frequency chirp Δ*ν*(*t*) and the associated harmonic distortion and RIN. We also characterize the modulation response to two-tone sinusoidal modulation and calculations of the corresponding intermodulation distortion and SFDR.

#### 4.3.1. Single-Tone Modulation

**Figure 3:**
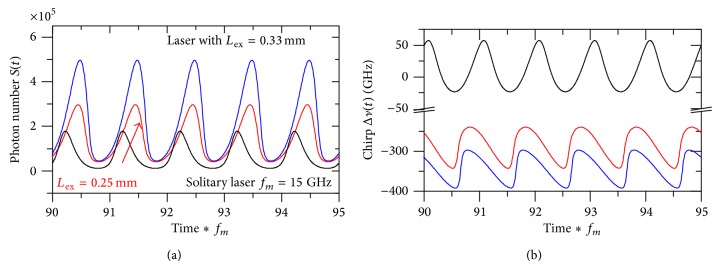
Modulation characteristics of the laser under OFB with *L*_ex_ = 0.25 mm and *f*_*m*_ = 55.8 GHz, with *L*_ex_ = 0.30 mm and *f*_*m*_ = 46 GHz, and the solitary laser with *f*_*m*_ = 15 GHz: (a) modulated waveform *S*(*t*) and (b) frequency chirp Δ*ν*(*t*).

Figures 3(a) and 3(b) plot the time variations of the photon number *S*(*t*) and the associated frequency chirp Δ*ν*(*t*), respectively, of the mm-frequency modulated laser signals that correspond to Figure 2. For comparison, the signal characteristics of the solitary laser modulated at *f*_*m*_ = *f*_*r*_ are also plotted in the figures.  Figure 3(a) indicates that the modulated signals are of the period-1 type oscillation. The oscillation amplitude of the modulated signal when *L*_ex_ = 0.30 mm is larger than that when *L*_ex_ = 0.25 mm, which agrees with the higher IM enhancement shown in Figure 2(a). The oscillation frequency of the signals is 15, 55.8, and 46 GHz of the solitary laser and laser with external cavities of lengths *L*_ex_ = 0.25 and 0.30 mm, respectively. The modulated signals under OFB are almost sinusoidal, whereas the modulated signal of the solitary laser with *f*_*m*_ = *f*_*r*_ tends to be clipped. The fast Fourier transform (FFT) analysis of these signals indicates that the mm-wave modulated signals have 2nd order harmonic-order distortion of 2HD = –7.4 and –6.0 dB when *L*_ex_ = 0.25 and 0.30 mm, respectively, which are lower than that of the solitary laser (–2.87 dB). The 2HD is calculated as [35]

(12) $$\begin{array}{c} 2\text{HD}=10\;\log_{10}\frac{a_{2}}{a_{1}}, \end{array}$$

where *a*_1_ and *a*_2_ are the FFT components at the fundamental frequency *f*_*m*_ and its second order harmonic, respectively.

On the other hand, Figure 3(b) shows that both the mm-modulated lasers under strong OFB with *L*_ex_ = 0.25 mm and 0.30 mm are red-shifted and the depth of the frequency chirp Δ*ν*(*t*)|_max⁡_ is 103 and 95 GHz, respectively, while the 15 GHz-modulated solitary laser is blue shifted and Δ*ν*(*t*)|_max⁡_ = 57 GHz. These results are consistent with relative amplitudes of the FM response of the solitary laser in Figure 1 and the lasers under OFB with *L*_ex_ = 0.25 and 0.30 mm in Figure 2. The frequency red shift of the laser with enhanced IM response is because the strong OFB results in a decrease in the carrier number *N*(*t*) under the threshold level *N*_th_ [27], as indicated from the rate equation (3).

**Figure 4:**
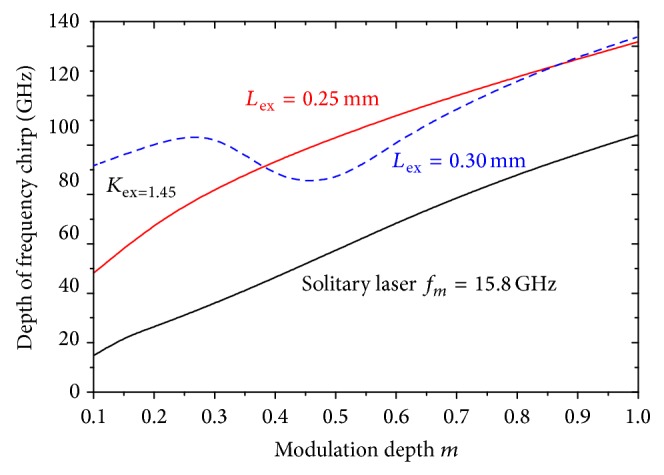
The frequency chirp Δ*ν*(*t*) associated with intensity modulation as a function of the modulation depth for both the ultrahigh frequency modulated laser under OFB with *L*_ex_ = 0.25 and 0.30 mm and the solitary laser with *f*_*m*_ = *f*_*r*_.

The comparison of the depth of the frequency chirp Δ*ν*(*t*)|_max⁡_ between the modulated signal of solitary laser and those of the laser under OFB when *L*_ex_ = 0.25 and 0.30 mm is examined over a wide range of the modulation index *m* as given in Figure 4. Figure 4 shows that the depth of the frequency chirp Δ*ν*(*t*)|_max⁡_ increases almost linearly with the increase in *m* for the three cases. Over the entire range of *m*, Δ*ν*(*t*)|_max⁡_ of the 46 and 55.8 GHz-modulated lasers under OFB are almost 30–70 GHz and 40 GHz larger than that of the 15 GHz-modulated solitary laser.

**Figure 5:**
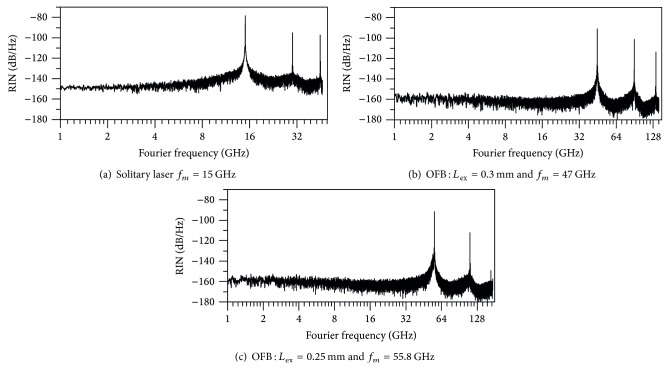
Frequency spectra of RIN of (a) solitary laser modulated at *f*_*m*_ = *f*_*r*_, (b) laser under OFB with *L*_ex_ = 0.30 mm and *f*_*m*_ = 46 GHz, and (c) laser under OFB with *L*_ex_ = 0.25 mm and *f*_*m*_ = 55.8 GHz.

We examine the noise content of the modulated signal in terms of the spectral characteristics of RIN. In Figure 5, we plot the frequency spectra RIN of the mm-frequency modulated laser under strong OFB.  Figure 5(a) plots the RIN spectrum of the solitary laser when modulated at the relaxation frequency *f*_*m*_ = *f*_*r*_, while Figures 5(b) and 5(c) plot the RIN spectra of the 46 GHz and 55.8 GHz-modulated laser under OFB from external cavities with *L*_ex_ = 0.30 mm and 0.25 mm, respectively. The figures show that the RIN spectra have sharp peaks at the corresponding modulation frequency *f*_*m*_ and at the higher harmonics. The low-frequency part of the RIN spectrum is almost flat (white noise). The level of this low-frequency noise, LF-RIN, is an inverse measure of the signal to noise ratio of the modulated signal [36]. The figures indicate that LF-RIN of the mm-frequency modulated laser is almost one-order of magnitude lower than that of the 15 GHz-modulated solitary laser. That is, compared with the solitary laser modulated at the relaxation frequency, the laser subjected to strong OFB and modulated within the mm-frequency band with enhanced modulation characterized by a signal with lower amplitude of intensity fluctuations.

#### 4.3.2. Two mm-Tone Modulation Characteristics

In the case of modulation with two mm-frequencies*f*_*m*1_ and *f*_*m*2_ with *f*_*m*1_ < *f*_*m*2_, the injection current *I*(*t*) in (1) is given by

(13) $$\begin{array}{c} I=I_{b}\left\{1+m\left[\cos\left(2\pi f_{m1}t\right)+\cos\left(2\pi f_{m2}t\right)\right]\right\}. \end{array}$$

**Figure 6:**
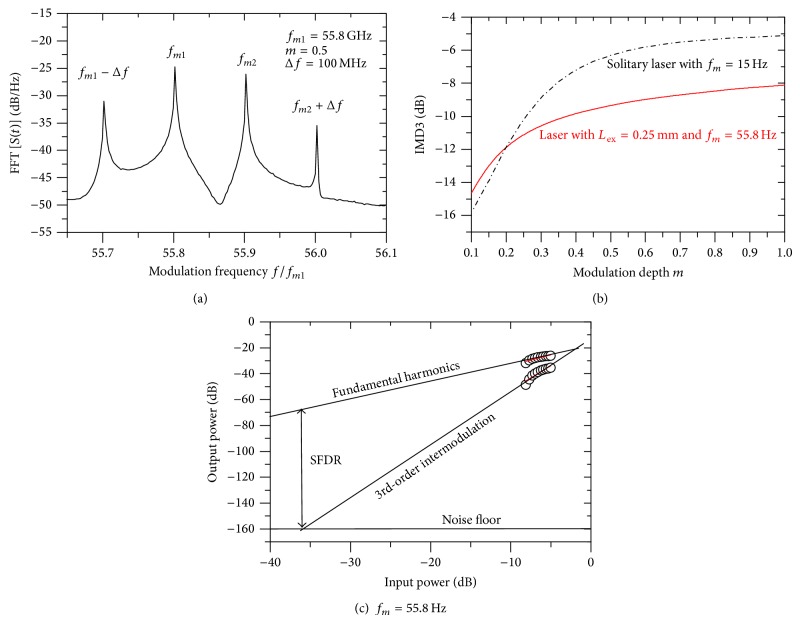
Characteristics of two-tone modulation with *f*_*m*1_ = 55.8 GHz and Δ*f* = 100 MHz: (a) FFT power spectrum showing the intermodulation components at *f*_*m*1_ − Δ*f* and *f*_*m*2_ + Δ*f*, (b) influence of modulation depth *m* on the IMD3, and (c) SFDR determination, SFDR = 73 dB/Hz^3/2^.

Such two-tone modulation is important for several applications, such as multichannel RF-frequency division multiplexed transmission of analog or microwave signals [37]. However, this modulation is often associated with intermodulation distortion, which occurs when the nonlinearity of the laser causes undesired outputs at sum and difference frequencies. The IM3 of two closely spaced carrier frequencies (at *f*_2_ and *f*_2_ + Δ*f*) is of particular interest [5].  Figure 6(a) plots the two-tone modulation response of the laser under OFB when modulated at *f*_*m*1_ = 55.8 GHz and *f*_*m*2_ + Δ*f* using the frequency spacing Δ*f* = 10 MHz. The figure corresponds to the modulation depth *m* = 0.5. The figure shows appearance of the 3rd order intermodulation components at *f*_*m*1_ − Δ*f* and *f*_*m*2_ + Δ*f* in addition to the fundamental harmonics at *f*_*m*1_ and *f*_*m*2_. IMD3 is defined as the ratio, in dB, of the amplitude of the third order intermodulation component to that of the fundamental component [26]:

(14) $$\begin{array}{c} \text{IMD}3=10\;\log_{10}\frac{a_{f_{m2}+\Delta f}}{a_{f_{m2}}}. \end{array}$$

Figure 6(b) plots IDM3 as a function of the modulation depth *m*. The figure shows that IMD3 increases with the increase in *m*. The slope of such increase is large in the regime of small-signal modulation and decreases with the increase in *m*. The figure indicates that IMD3 ranges between −14.5 and −8 dB. In the figure, we also compare the IMD3 values with those of the solitary laser when modulated at the carrier-photon resonance frequency *f*_*m*1_ = *f*_*r*_. As shown in the figure, IMD3 of the solitary laser is little lower than that of the laser under strong OFB up to *m* = 0.2. For modulation with larger signals, IMD3 of the solitary laser becomes larger and the differences reache 3 dB when *m* = 1.0. These values of IMD3 limit the performance of the laser-based RoF links. This limitation is measured by SFDR. SFDR is determined by three quantities, namely, the power of the fundamental frequency component, noise floor, and the power in the IMD3 component [26]. The noise floor is determined from the RIN spectrum of the free-running laser when *I*_*b*_ = 5*I*_th_.  Figure 6(c) plots the output powers of the fundamental signal and the 3rd order intermodulation power and the noise floor versus the input electrical power. A linear fit is made to the plotted data and the SFDR is extracted as SDFR = 83 dB/Hz^3/2^. The calculated value of SFDR of the solitary laser when modulated at the *f*_*m*_ = *f*_*r*_ was found to be 2 dB/Hz^3/2^ lower.

## 5. Conclusions

We presented the modeling of mm-frequency modulation characteristics of semiconductor lasers under strong OFB. The study was based on the theoretical modeling fully handling the strong OFB regime as time delay of laser light due to round-trips in the external cavity. We analyzed the signal distortions and noise associated with both single- and two-tone modulations. We show that the enhanced IM response under strong OFB is due to photon-photon resonance resulting from coupling of oscillating modes in the external cavity. When the beating frequency of these coupled modes matches the frequency of the modulating electrical signal, the modulation response reveals resonance over a narrow-frequency band. A key parameter to achieve this mm-wave photon-photon resonance is to modulate the laser when it keeps stable operation in CW under strong OFB, where the injected delay light becomes in phase with the optical field in the laser cavity. Within this mm-frequency passband with enhanced IM response, the laser emits period-1 oscillations with low harmonic distortion. The LF-RIN level increases very little with the increase in the modulation depth. Under modulation with two adjacent mm-frequencies, IMD3 increases with the increase in the modulation index *m*, ranging between −14.5 and −8 dB. Compared with the solitary laser modulated at the relaxation frequency, the present modulated signal was shown to have 10 dB/Hz lower RIN and 2 dB/Hz^3/2^ higher SFDR.

## Figures and Tables

**Table 1:** Definition and numerical values of the solitary high-speed laser parameters.

Symbol	Definition	Value
*λ*	Wavelength	1.55 *μ*m
*V*	Active layer volume	3 × 10^−17^ m^3^
*v*_*g*_	Group velocity	8.33 × 10^7^ m/s
*L*_*D*_	Active layer length	120 *μ*m
*a*	Differential gain coefficient	8.25 × 10^−12^ m^2^
*N*_*g*_	Carrier number at transparency	3.69 × 10^7^ m^−3^
α	Linewidth enhancement factor	3.5
Γ	Confinement factor	0.15
*τ*_*p*_	Photon lifetime	1.69 ps
*τ*_*s*_	Spontaneous emission lifetime	776 ps
*R*_*f*_	Front facet reflectivity	0.2
*R*_*b*_	Back facet reflectivity	0.6
*β*_*sp*_	Spontaneous emission factor	3 × 10^−5^
*ε*	Nonlinear gain suppression factor	2.77 × 10^−23^ m^3^
